# Clinical Characteristics of Major Depressive Disorder in Children: Experience from the Child Psychiatry Department of Casablanca University

**DOI:** 10.1192/j.eurpsy.2026.11137

**Published:** 2026-07-31

**Authors:** H. Nemrany, H. El oualidi, S. Benkirane, S. El hormi, L. Rachidi

**Affiliations:** Service universitaire de pédopsychiatrie, CHU Ibn Rochd, casablanca, Morocco

## Abstract

**Introduction:**

Major depressive disorder (MDD) in children represents a significant public health concern and a persistent diagnostic challenge in psychiatry. Long underestimated, it remains difficult to identify due to the variability and subtlety of clinical manifestations.

**Objectives:**

To outline the epidemiological profile and describe the clinical characteristics of children diagnosed with MDD in child psychiatry services.

**Methods:**

A retrospective analytical study was conducted in the Child Psychiatry Department of Ibn Rochd Hospital, Casablanca, over two years (August 2021–August 2023). The study included 380 children aged 6–12 years diagnosed with MDD. Sociodemographic, clinical, comorbid, and therapeutic data were analyzed.

**Results:**

A female predominance was observed (54.2%; sex ratio 0.84). The mean age was 8.9 years, with the 8–12 age group being the most represented. Sleep disturbances were the most common symptom (51.3%). Depression was mild in 40.8% of cases and severe in 21.3%. Suicidal ideation occurred in 13% of cases, while suicide attempts were reported in 2%. Frequent comorbidities included learning disorders (52.8%), generalized anxiety disorder (51.8%), obsessive-compulsive disorder (48.9%), and separation anxiety disorder (46%). Outpatient management was predominant (80%), while 20% of children required hospitalization. Psychotherapy alone was prescribed in 85.1% of cases, while 14.9% received combined treatment. Sertraline was used in 97.5% of pharmacological interventions.

**Conclusions:**

Childhood depression is a frequent and serious clinical condition. This study highlights its epidemiological and clinical patterns, along with common psychiatric comorbidities. Our findings underscore the need for early detection, tailored therapeutic strategies, and educational tools to optimize management and improve prognosis in this vulnerable population.

**Disclosure of Interest:**

None Declared

